# Dynamic network impairments underlie cognitive fluctuations in Lewy body dementia

**DOI:** 10.1038/s41531-022-00279-x

**Published:** 2022-02-17

**Authors:** Elie Matar, Kaylena A. Ehgoetz Martens, Joseph R. Phillips, Gabriel Wainstein, Glenda M. Halliday, Simon J. G. Lewis, James M. Shine

**Affiliations:** 1 Central Clinical School, Faculty of Medicine and Health, University of Sydney, Sydney, NSW Australia; 2 Forefront Parkinson’s Disease Research Clinic, Brain and Mind Centre, University of Sydney, Sydney, NSW Australia; 3 Forefront Research Team, Brain and Mind Centre, University of Sydney, Sydney, NSW Australia; 4 Department of Kinesiology, University of Waterloo, Waterloo, ON Canada; 5 School of Social Sciences and Psychology, Western Sydney University, Sydney, NSW Australia; 6 Centro de Investigaciones Médicas, Pontifical Catholic University of Chile, Santiago, Chile

**Keywords:** Diagnostic markers, Neurodegeneration, Dementia

## Abstract

Cognitive fluctuations are a characteristic and distressing disturbance of attention and consciousness seen in patients with Dementia with Lewy bodies and Parkinson’s disease dementia. It has been proposed that fluctuations result from disruption of key neuromodulatory systems supporting states of attention and wakefulness which are normally characterised by temporally variable and highly integrated functional network architectures. In this study, patients with DLB (*n* = 25) and age-matched controls (*n* = 49) were assessed using dynamic resting state fMRI. A dynamic network signature of reduced temporal variability and integration was identified in DLB patients compared to controls. Reduced temporal variability correlated significantly with fluctuation-related measures using a sustained attention task. A less integrated (more segregated) functional network architecture was seen in DLB patients compared to the control group, with regions of reduced integration observed across dorsal and ventral attention, sensorimotor, visual, cingulo-opercular and cingulo-parietal networks. Reduced network integration correlated positively with subjective and objective measures of fluctuations. Regions of reduced integration and unstable regional assignments significantly matched areas of expression of specific classes of noradrenergic and cholinergic receptors across the cerebral cortex. Correlating topological measures with maps of neurotransmitter/neuromodulator receptor gene expression, we found that regions of reduced integration and unstable modular assignments correlated significantly with the pattern of expression of subclasses of noradrenergic and cholinergic receptors across the cerebral cortex. Altogether, these findings demonstrate that cognitive fluctuations are associated with an imaging signature of dynamic network impairment linked to specific neurotransmitters/neuromodulators within the ascending arousal system, highlighting novel potential diagnostic and therapeutic approaches for this troubling symptom.

## Introduction

Lewy body dementia, which comprises Dementia with Lewy bodies (DLB) and Parkinson’s disease dementia (PDD), is a common neurodegenerative disease with a higher morbidity, socioeconomic cost and caregiver burden relative to other dementias^1^. Marked and spontaneous alterations in consciousness and attention, known as cognitive fluctuations, are present in up to 90% of DLB patients and are recognised as a core diagnostic feature^2,3^. Behaviourally, they can range from an impairment of consciousness with reduced responsiveness to more subtle variability in the performance of attentional tasks^4^. Despite its frequency and clinical relevance in DLB, objective biomarkers for cognitive fluctuations are lacking and our understanding of their neuroanatomical substrates remains limited.

Available evidence points to several key features relating to the mechanisms of fluctuations that emphasise the importance of considering both the spatial and temporal properties of the brain^5^. Indeed, previous approaches in DLB relying only on structural markers or time-averaged functional imaging measurements have yielded varied findings regarding the contribution of individual regions or networks^6–9^. Dynamic functional connectivity, which aims to capture meaningful time-dependent variations in connectivity that are often missed in static network models, is emerging as a promising tool for understanding the neural basis of attentional and cognitive disturbances in Lewy body diseases^10–14^.

Dynamic connectivity approaches in healthy individuals have shown that an important feature of the conscious and attentive brain is the ability to dynamically explore a diverse repertoire of network configurations^15,16^. At a macroscopic level, these network reconfigurations can be depicted by flexible transitions along a continuum from highly segregated information processing via short range connections between regions at one end and long-range integrated activity between many regions at the other^17^. Highly integrated and temporally variable states have been shown to correlate with awareness and consciousness^15,18–20^, and with better performance on cognitive tasks^16,21,22^. Such network-level characteristics have been linked to the influence of distinct neuromodulatory neurotransmitters forming part of the ascending reticular activating system^23^. Specifically, acetylcholine and noradrenaline, which facilitate states of wakefulness and alertness^24^, have also been implicated in the interplay between effective network level segregation and integration respectively^16,23^, and involvement of these systems has been hypothesised to be an important component of the pathophysiology underlying cognitive fluctuations in DLB^5^.

In this study, we set out to test the hypothesis that cognitive fluctuations in DLB may be linked to the perturbation of specific neurotransmitter/neuromodulatory systems, which would be reflected by specific dynamic changes in brain topology measured using resting state fMRI. We show that compared to healthy age-matched controls, DLB patients demonstrate a functional signature of reduced network-integration and low temporal variability that could be directly related to both subjective and objective measures of cognitive fluctuations. Uniquely, we also explored whether these alterations in neural connectivity could be related to specific neurotransmitters/neuromodulators. By correlating our topological fMRI connectivity measures with data from established receptor gene expression maps, we confirm an explicit link between noradrenaline and acetylcholine neurotransmission and the pathophysiology of altered brain dynamics that underlie the cognitive fluctuations observed in DLB.

## Results

### Reduced temporal variability of brain states in DLB

**Fig. 1:**
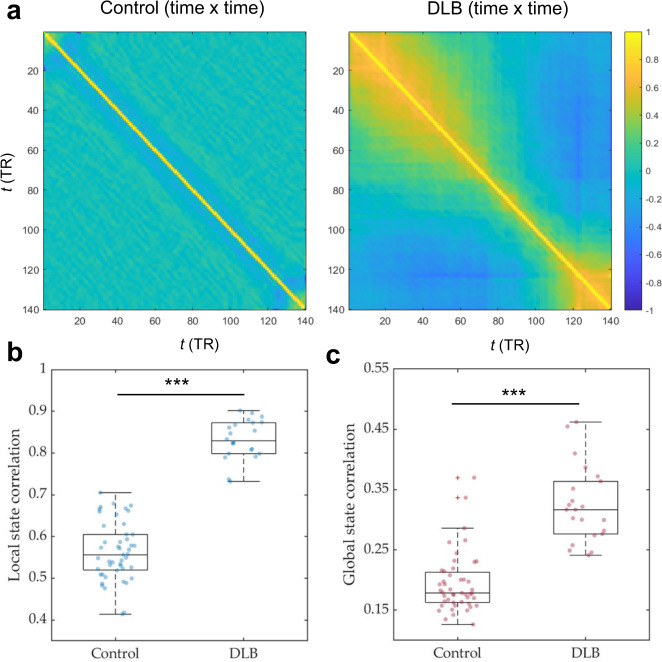
DLB is characterised by more invariant dynamics at a macroscopic level which is defined by higher intertemporal correlation of brain connectivity state configurations. This is visually depicted by the time-to-time correlation matrices (**a**) for controls (left panel) and patients with DLB (right panel) and displays the coefficient of correlation of the functional connectivity network between any two time points averaged across all regions. This information is captured quantitatively by the summary statistics displayed in the boxplots below. **b** Patients with DLB demonstrate significantly increased local Similarity (S_L_; correlation of network state between all contiguous time points) compared to control subjects. **c** Patients with DLB also exhibit higher global similarity (S_G_; network state correlations across all time points) relative to controls. ***P < 0.001, permutation-testing.

We evaluated the macroscopic dynamics of the brain by investigating the degree of reconfiguration between different connectivity patterns over time (Fig. 1a). DLB patients were found to have a more invariant (stationary) brain-state configuration, demonstrated by significantly higher correlations of whole brain activation patterns between any two contiguous time points (local similarity, *S*_*L*_) relative to controls (0.83 ± 0.07 versus 0.56 ± 0.07 respectively; *P* < 0.001, Cohen’s *d* = 1.9; Fig. 1b). We also found that the diversity of observed brain activation patterns was also reduced in DLB, as demonstrated by significantly higher global state-to-state correlation (*S*_*G*_) across the imaging period compared to controls (0.32 ± 0.06 versus 0.19 ± 0.05; *P* < 0.001, Cohen’s *d* = 1.6; Fig. 1c). To support our findings, we found no significant differences in either local or global similarity between our study’s control group and a validation sample of 477 controls (*S*_*L*_ = 0.48 ± 0.1, *P* = 0.37; *S*_*G*_ = 0.21 ± 0.07, *P* = 0.46; two-sided independent samples *t* test).

### DLB is characterised by segregated network topology

**Fig. 2:**
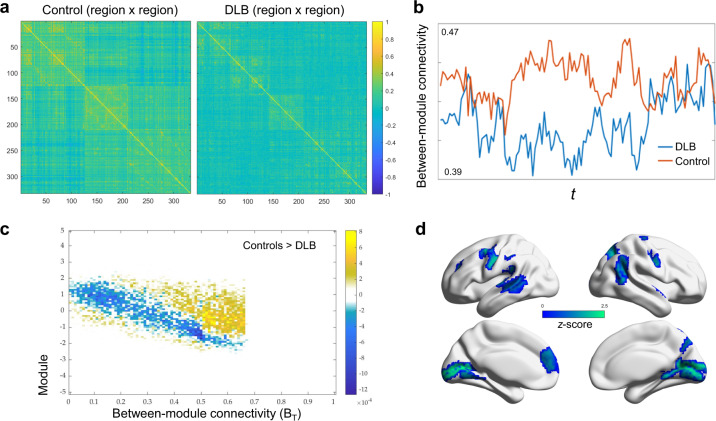
DLB characterised by less integrated dynamic network topology. **a** Time averaged connectivity matrices derived from multiplication of temporal derivatives of activation time series demonstrate reduced long-range functional connections and more segregated, modular structure in DLB compared to controls. **b** Time varying participation coefficients also demonstrate prolonged periods of reduced time-resolved participation (between-module connectivity) between DLB and control over duration of scan (representative region in visual cortex shown). **c** The cartographic profile of the network demonstrating higher network segregation in patients with DLB, as demonstrated by a greater number of regions over time (reflected by higher intensity on the histogram) occupying states of lower between-module connectivity (leftward shift) and equivalent or higher within module connectivity. **d** Topographic mapping of reduced participation in DLB demonstrates prominent involvement of medial, frontal, parietal and occipital regions.

Overall, DLB participants demonstrated a less integrated (more segregated) functional network topology compared to the control group (time-averaged mean *B*_*iT*_ across all regions; *P* < 0.001; Fig. 2a–c). At the regional level, these differences were especially driven by significantly reduced integration in the precuneus, posterior cingulate, parietal, insular, anterior temporal and medial frontal areas (Fig. 2d). These regions of reduced integration represent nodes within several putative networks including the dorsal and ventral attention networks, as well as the sensorimotor, visual, cingulo-opercular and cingulo-parietal networks. Using *k*-means clustering (*k* = 2), we found the proportion of time that any given region spent in a more segregated state (pertaining to the cluster of lower participation, *B*_*T*_) to be significantly greater in DLB (mean time spent in a ‘segregated’ state across all regions: 0.33 ± 0.03) compared to controls (0.11 ± 0.01; *P* < 0.001, multiple regression; Cohen’s *d* = 1.3). Within the DLB group specifically, we also found that proportion of time spent in a segregated state was significantly greater in participants with more severe fluctuations (*n* = 11) compared to those without/less severe fluctuations (*n* = 11) as stratified using conventional carer-reported fluctuation severity (CAF cut-off score = 4^25,26^; *U* = 87.00; *P* = 0.04). The relationship between topology and objective measures of fluctuations is explored further below.

### DLB regions exhibit more unstable modular switching

**Fig. 3:**
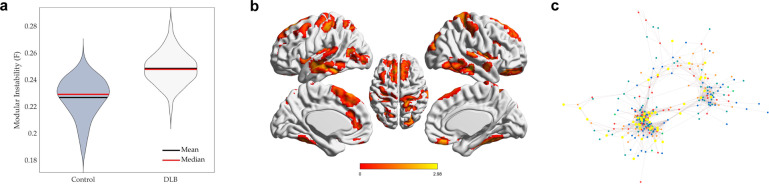
DLB is characterised by increased modular instability. **a** DLB regions had significantly increased modular instability (flexibility) relative to controls (*P* < 0.001). **b** Areas of increased flexibility (FDR < 0.1) included boundary regions between modules as well as distributed diffusely throughout inferior and middle temporal, left medial frontal and post-central parietal regions. **c** Force-directed plot of areas of significantly increased instability (yellow dots; FDR < 0.05) in relation to communities (shown in smaller coloured dots – green, blue, red) demonstrates these regions are spread diffusely throughout different modules both embedded in high-degree communities as well as at the junction points between modules.

Having established a highly segregated and stationary functional architecture in DLB patients at a macroscopic scale relative to controls, a measure of modular instability/flexibility was calculated to see whether regions were likely to remain in the same communities or switch between different communities whilst maintaining a macroscopically segregated architecture. Using pairwise comparisons, we found that regions were more likely to have unstable assignments (increased flexibility) in DLB compared to controls (mean flexibility DLB: 0.32 ± 0.07; Controls: 0.26 ± 0.17; *P* < 0.001; Cohen’s *d* = 0.46, Fig. 3a). Individual regions of significantly increased flexibility shown in Fig. 3b included predominantly ventro and dorso-medial frontal cortex, as well as inferior temporal and dorsal parietal regions, which subserve frontoparietal, default mode and auditory networks.

### Relationship between macroscopic dynamics and attentional performance in DLB

**Fig. 4:**
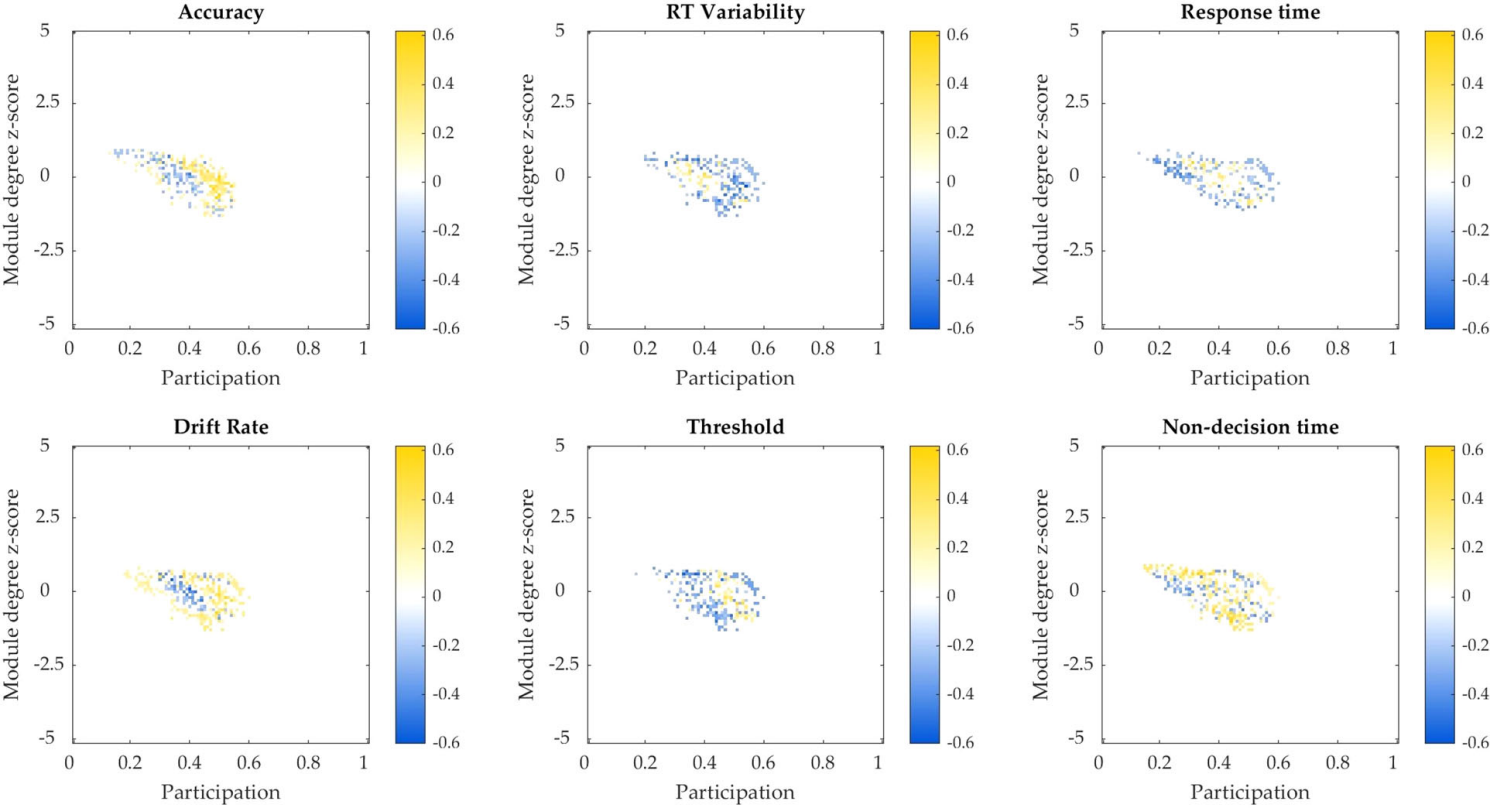
Correlation between the cartographic topological profile and attentional parameters from the SART. The cartographic profile is a histogram mapping of the functional activity of each parcellated region of the brain according to the topological dimensions of between-module connectivity (participation) and within-module connectivity (module degree *z* score). Correlations displayed for regions with FDR < 0.2. Patterns of increased integration can be seen to positively map to higher accuracy and drift rate, while inversely mapping (negative correlation) with variability in response time (RT). Colour bar denotes Pearson’s correlation coefficient. RT response time.

We next explored whether the changes in dynamical and topological measures seen in DLB related to objective measures of attentional performance in these patients. In DLB participants, we found a statistically significant positive correlation between local similarity (*S*_*L*_) and the response time variability (standard deviation of response time) on the task (*ρ* = 0.43, *r*^*2*^ = 0.2; *P* = 0.04). Fitting a drift diffusion model to the data (Supplementary Table 1), we also found a significant negative correlation between local similarity (*S*_*L*_) and drift rate (*ρ* = −0.41, *r*^*2*^ = 0.2; *P* = 0.04). We did not find a similar association with global cognitive performance (MMSE; *P* = 0.11), nor did we find any significant correlations between attentional performance and global similarity, or time-averaged imaging measures (modularity, participation and module-degree z-score) when treated independently (Supplementary Table 1). However, correlation between attentional measures and brain topology using the cartographic profile of each individual (the relative distribution of highly integrated and segregated regions during the imaging period) confirmed that more regions in integrated states (rightward displacement) were positively correlated with accuracy and drift rate, but inversely correlated with RT variability (Fig. 4). Importantly, similar patterns (though weaker) were also observed for subjective measures of fluctuations including the CAF severity (especially duration component) and OFS total score – with distinctly higher scores correlating positively with regions of segregation and negatively with areas of increased integration (Supplementary Fig. 1).

### Correlation between DLB topology and neurotransmitter/neuromodulator receptor gene expression

**Fig. 5:**
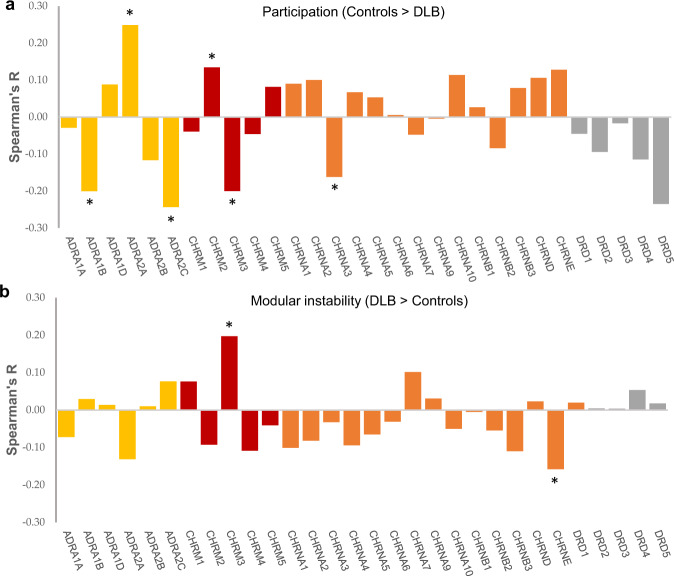
Spearman correlations between regional cortical expression of adrenergic, cholinergic (muscarinic and nicotinic), and dopaminergic receptors and difference in topological parameters seen between controls and DLB. **a** Correlation between gene expression and difference in participation (integration) between controls and DLB showed a significant relationship between differential expression of adrenergic and cholinergic receptors with later confirmation with statistical testing showing significant differences for *ADRA2A* and *CHRM2*. **b** Correlation between gene expression and difference in modular instability (flexibility) (DLB > Controls) highlighting significant relationships with cholinergic receptor expression, later confirmed for *CHRM3*. See Supplementary Table 2 for gene names. *FDR < 0.1.

To examine the neuromodulatory underpinnings of the topological disturbances seen in DLB, we investigated the relationship between maps of neurotransmitter/neuromodulator receptor gene expression (Supplementary Table 2) and the regions of significant difference in topological measures. We initially performed a whole-brain analysis of the correlations between regional expression of noradrenergic, dopaminergic receptors and cholinergic receptors and mean regional difference between measures of participation and modular instability between controls and DLB (Fig. 5). Results of this exploratory analysis showed significant correlations between adrenergic and cholinergic (muscarinic) receptors and differences in regional participation (FDR < 0.1). Confirmatory analysis comparing between areas only of significantly reduced participation between DLB and controls found significantly higher mean expression of *ADRA2A* (alpha-2a adrenergic receptor; *P* = 0.003, independent samples *t* test) and *CHRM2* (muscarinic acetylcholine receptor M2; *P* = 0.008) compared to all other regions that were not significantly different, suggesting that regions that were segregated may have been so due to insufficient recruitment of activity within these receptor distributions. Whole brain analysis showed differences in modular instability correlated with changes in mean expression of muscarinic and nicotinic cholinergic receptors which were confirmed in subsequent analysis to show significance for increased expression for *CHRM3* (*P* = 0.01). No significant relation between dopaminergic receptor expression and the topological parameters were seen in our cohort.

## Discussion

Recent work using resting state fMRI in humans and non-human primates has suggested that in addition to high levels of global integration, conscious wakefulness is marked by the ability to dynamically explore multiple repertoires of brain configurations^15,19^. This temporal variability exists at multiple levels of neuronal organisation^27^ and is lost under conditions of reduced consciousness^15,19,28,29^. Disturbances in consciousness and alertness are characteristic of the phenomenon of fluctuating cognition of DLB. In this study, we were able to define simple metrics that capture the moment-to-moment temporal variability using resting state fMRI data that differentiates DLB patients from controls. The finding that local and global similarity is significantly increased in DLB implies that DLB brains are more fixed in time, with slower transitions between different configurations and suggests a reduced repertoire of brain configurations over the entire scanning session. Importantly, we found the loss of temporal variability is linked to the phenomenon of fluctuations by showing a significant and specific correlation between local similarity and response time variability and drift rate extracted from an objective attentional task.

The ability to adapt to changing cognitive demands through a balance of integrated and segregated information processing has also been suggested as an essential property of consciousness and normal brain function^17^. Using graph-theoretical measures, we found that the DLB connectivity pattern is characterised by reduced integration (Fig. 2). This was distributed across nodes of task-positive networks that have been shown empirically to support several attention-related functions. The dorsal attention network, for instance, has been proposed to underlie selective attention, particularly in relation to visuospatial tasks^30,31^ and dysfunction within the dorsal and ventral attentional networks has been demonstrated in DLB using event-related fMRI^32^. The cingulo-opercular network has been shown in task-based fMRI studies to play an important role in tonic alertness, which is an important component of task used in this study^33^. Interestingly, pathology and dysfunction in the cingulate cortex and its associated networks have been reported in DLB^4,34^.

The relationship between network topology and fluctuations is suggested by the finding that the degree of regional segregation correlated positively with response time variability and negatively with drift rate on an attentional task. Response time variability has long been regarded as an objective diagnostic marker of fluctuations^2,35^. Drift rate accounts for the effects of random influences such as individual motor impairments to response times^36^. Thus, findings of reduced drift rate in DLB reinforces the intrinsic relationship between our topological measures here and cognition specifically. Analyses using subjective reporting of fluctuations, though weaker, were aligned with the objective measures (Supplementary Fig. 1). Together, these findings confirm that disruption of long-range intermodular cortical connections and more rigid functional architecture differentiate DLB from controls and relate to attentional fluctuations. This provides convergent support for the assertions of recent studies in Parkinson’s disease dementia and DLB which have characterised the functional dynamics using alternative analytical approaches^10,12,14^.

We completed our characterisation of the functional topology of the DLB brain by showing that the assignment of any region to a specific module in DLB was more unstable than controls, a finding also seen in patients with Parkinson’s disease who are off their dopaminergic treatment^11^. While this finding may seem counterintuitive in light of the increased stationarity seen in DLB, this can be reconciled with the above findings by proposing that in addition to a reduced amount of integration in DLB, there is also loss of stable and effective segregation necessary for efficient information processing.

The balance of integration and segregation have been shown to be distinctly related to the neuromodulatory roles of noradrenaline and acetylcholine, respectively^23,37^. These are an important component of the ascending arousal system responsible for maintaining consciousness and regulating transitions from sleep to wakefulness^24,38^. In our exploratory analysis, we were able to show that regional differences in integration and modular stability (effective segregation) correlates significantly with the expressional patterns of cholinergic and adrenergic receptors. Subsequently, we found regions of reduced integration in DLB were specifically associated with high levels of expression of the cholinergic muscarinic-2 receptor (CHRM2) and the adrenergic-2α receptor (ADRA2A). Interestingly, among many genes investigated in humans in relation to general intelligence, the association of *CHRM2* with cognitive ability is one of the most replicated findings^39,40^. Meanwhile, polymorphisms in *ADRA2A* have been shown in several studies to be linked to attention deficit hyperactivity disorder (ADHD)^41^ and the classes of drugs used to treat attention disorders are known to act as direct agonists on the α2-adrenoceptor^42^. Expression of the muscarinic cholinergic 3 receptor (*CHMR3*) was found in our study to be significantly different in regions of increased modular instability. Although studies are few, CHRM3 expression has been shown to be altered in the prefrontal cortex of patients with bipolar disorder^43^ and linked to abnormal thalamo-frontal connectivity in patients with schizophrenia^44^. The signal for the role of the cholinergic and noradrenergic system in the pathophysiology of fluctuations in DLB is consistent with pathological studies. Cholinergic cell involvement is well recognised and linked to cognitive symptoms in DLB^45–48^(3,65-68). Pathological involvement of noradrenergic structures, such as the locus coeruleus, have also been reported in Lewy body disorders^49–51^, but until now the link to fluctuations in DLB has remained mostly speculative. Confirmation of our findings would provide a rationale for investigating the benefit of therapies targeting not only the cholinergic system (currently the mainstay of treatment) but also concurrently the noradrenergic system in DLB. This could be achieved through the repurposing of medications such as noradrenaline reuptake inhibitors and adrenoceptor agonists currently used to treat depression and ADHD.

A previous study, although aligned in principle, was unable to find a link between dynamic connectivity measures and subjective scoring of fluctuations^13^. Subjective scales of fluctuations were not designed to accurately capture variance in attentional disturbances but rather were designed with diagnostic purposes in mind^52^. Complementing the use of questionnaires with objective tests of attention here was able to yield more robust relationships with imaging measures. In addition, the use of continuous metrics in this study to characterise the macroscopic dynamics is likely to be more physiologically representative and sensitive in capturing the variance of temporal dynamics than artificial divisions of discrete states derived from commonly used clustering-methods. Nonetheless, the reproducible signature of a segregated and temporally invariant brain between studies is reassuring and advocates strongly for its consideration as a diagnostic tool in place of the subjective measures currently being used. We suggest the continuous measures used here, which are amenable to sensitivity and specificity analyses, may be especially useful in prodromal populations (such as in patients with mild cognitive impairment or idiopathic REM sleep behaviour disorder) in which changes in macroscopic dynamics may occur gradually and continuously and could be used as indicators of disease progression.

Several limitations of our study should be noted. First, our protocol comprised a relatively short scanning time which was chosen to minimise the risk of participants falling asleep in the scanner. Given the logistical difficulties of scanning patients with dementia, the finding of clear differences in this timeframe suggests it is a potentially tractable diagnostic tool. Studies exploring the utility of longer scanning times, controlling for sleep may be required in evaluating the diagnostic utility in prodromal populations where the changes may be more subtle. Second, although our study was able to detect salient differences within a well-phenotyped single centre cohort of patients with DLB, our sample size may have constrained statistical significance to correlations with larger effect sizes and limited the generalisability of our results. Future analyses using large cohorts combining multicentre data will be the necessary next step to validate our findings. Third, limitations in the use of gene expression atlases should also be noted, including inherent measurement variability of transcriptomic data, donor-specific variability, and spatial autocorrelation (for a review, see^53^). Fourth, while withdrawal of cholinergic medications was not feasible in our DLB patients who manifested fluctuations even on cholinergic medications, it is possible that the magnitude or direction of the dynamic connectivity results may have been attenuated. Although not direct agonists, it is possible that regions subserved more by cholinergic receptors with greater binding affinity to acetylcholine may have been less affected. Future testing in drug-naïve patients may increase power in detecting a significant effect for these regions. Finally, concurrent testing of attention in the scanner in early/prodromal DLB will also be important for exploring in more detail the timescale correlations between the ascertained functional dynamic signatures and fluctuations within attentional tasks.

By characterising the topology and dynamics of resting state connectivity in patients with DLB, we provide a plausible functional imaging signature of cognitive fluctuations that converges well with recent data and theory. Uniquely, we show the ability of such measures to connect with the underlying biology by implicating specific neurotransmitter/neuromodulator systems, and thus offering new therapeutic avenues. Longitudinal studies using these metrics will be essential to confirming its utility as a marker for tracking disease or symptom progression and response to therapy.

## Methods

### Participants

Twenty-five DLB participants were prospectively and consecutively recruited from a community dwelling population referred to a dementia and movement disorders clinic at the Brain and Mind Centre, University of Sydney. All participants underwent clinical assessment by a neurologist and were diagnosed as having probable DLB according to the 2017 consensus criteria^2^. Polysomnography was obtained from all patients and the presence of REM Sleep Behaviour Disorder (RBD) was determined according to the International Classification of Sleep Disorders-III criteria. Parkinsonism was diagnosed on examination by a movement disorders specialist (S.J.G.L) and quantified according to the revised Movement Disorders Society Unified Parkinson’s Disease Rating Scale (MDS-UPDRS)^54^. Visual hallucinations were determined on detailed independent interview with patient and caregiver. Severity of cognitive impairment was identified by screening with the Mini-mental state examination (MMSE;^55^). The presence of cognitive fluctuations was confirmed by detailed semi-structured interview with the patient and caregiver and the severity was rated using the Clinician Assessment of Fluctuations Scale (CAF) and One Day Fluctuation Scale (OFS;^25^). Patients were tested on stable doses (>1 month) of their cholinergic and/or dopaminergic medications. No patients were taking serotonin or noradrenergic reuptake inhibitors at the time of the study.

Forty-nine healthy controls were recruited to participate in the study. Control participants were recruited from the community via use of flyers, online advertising, word-of-mouth, email to previous study participants who have opted to be contacted for future studies and recruitment drives at local facilities. Control participants were excluded if they had a history of neurological or psychiatric disorders or had a prescription for psychoactive medications. Patients with DLB and healthy controls were matched for age and education. No participants were taking antipsychotics at the time of the study. The study was approved by the University of Sydney Human Research Ethics Committee with informed written consent obtained in accordance with the Declaration of Helsinki. Demographic details and clinical characteristics are reported in Table 1.

**Table 1:** Clinical characteristics.

	Control	DLB
*n*	49	22
Sex (M:F)*	14:35	18:4
Age	66.4 (8.5)	74.5 (6.1)
Education	13.5 (2.8)	12.0 (3.3)
MMSE*	28.9 (1.2)	22.7 (5.7)
Disease duration (years)	–	2.1 (1.3)
Motor: UPDRS-III	–	32.7 (15.5)
Hallucinations, *n* (%)	–	10 (45.5)
Fluctuations, *n* (%)	–	16 (72.7)
RBD, *n* (%)	–	18 (81.8)
DDE (mg)	–	150 (250)
Cholinergic dose (mg)^a^	–	5.2 (4.3)

Values presented as mean (standard deviation) unless otherwise specified as number of patients (percentages). Patients were matched on age and education. Groups significantly differed in sex and MMSE (*P < 0.05, Independent Samples *t* test). Disease duration recorded in years since diagnosis.

*UPDRS-III* Unified Parkinson’s Disease Rating Scale (Section III), *RBD* rapid eye movement sleep behaviour disorder, *DDE* dopamine dose equivalency.

^a^Refers to average rivastigmine—16 patients were on rivastigmine, 5 patients were not taking any cholinergic agents. The one subject taking donepezil 10 mg was excluded from the calculation.

### fMRI acquisition and pre-processing

Imaging was conducted on a General Electric 3 Tesla MRI. Whole-brain 3D T_1_-weighted sequences were acquired (200 slices, 1 × 1 mm^2^ in-plane resolution, flip angle 12°, slice thickness = 1 mm, echo time/repetition time = 2.7/7.1 ms). Resting T_2_*-weighted echo planar functional images were acquired in interleaved order (repetition time = 3 s, echo time = 36 ms, flip angle = 90°, 40 axial slices, field of view = 240 mm, raw voxel size = 3.75 × 3.75 × 3 mm thick, duration = 7 min). Patients were instructed to lie awake with their eyes closed and to let their minds wander freely without falling asleep. Patients were prompted prior to the fMRI sequence and interviewed after the sequence and scan to ensure these instructions were followed. Patients (*n* = 3) who reported falling asleep were excluded from the analysis.

Pre-processing of resting state fMRI images was conducted according to previously published methods^11^. Pre-processing was conducted using SPM12 (Statistical Parametric Mapping software; http://www.fil.ion.ucl.ac.uk/spm/software/). Scans were first slice-time corrected to the median slice in each repetition time, then realigned to create a mean realigned image, with measures of 6 degrees of rigid head movements calculated for later use in the correction of minor head movements. For quality assurance, each trial was analysed using ArtRepair and trials with a large amount of global drift or scan-to-scan head movements >1 mm were corrected using interpolation. None of the subjects included in this study demonstrated scan-to-scan head movements >3 mm (<1 voxel breadth). Images were normalised to the Echo Planar Image template and resampled to 3 mm isotropic voxels.

Temporal artefacts were identified in each dataset by calculating framewise displacement (FD) from the derivatives of the six rigid-body realignment parameters estimated during standard volume realignment^56^, as well as the root mean square change in BOLD signal from volume to volume (DVARS). Frames associated with FD > 0.25 mm or DVARS > 2.5% were identified. However, as no participants were identified with >10% of the resting time points exceeding these values, no sessions were excluded from further analysis.

Following artefact detection, nuisance covariates associated with the 12 linear head movement parameters (and their temporal derivatives), FD, DVARS, and anatomical masks from the CSF and deep cerebral white matter were regressed from the data using the aCompCor strategy^57^. In keeping with previous time-resolved connectivity experiments, a temporal bandpass filter (0.071 < f < 0.125 Hz) was applied to the data^11^. Given the importance of head motion in functional connectivity analyses, we compared the mean and standard deviation of framewise displacement across the entire resting state session between the two groups (controls, DLB)^56^. We found no correlations between connectivity measures below and head motion parameters.

Following the above steps, the mean time series was extracted from 333 predefined cortical parcels using the Gordon atlas^58^. Time-resolved functional connectivity was calculated between all 333 brain regions using the multiplication of temporal derivatives (MTD) metric within a sliding temporal window of 15 time points (∼45 s)^59^. Individual functional connectivity matrices were then calculated within each temporal window.

### Temporal correlations of brain state configurations

To estimate whether brain connectivity patterns (i.e., brain states) reconfigured at different temporal scales in the two groups, we calculated the Pearson’s correlation of the pattern of BOLD activity across the whole brain between every epoch (TR) of time. From this, we derived two complementary summary statistics:

1. Local similarity (*S*_*L*_): the correlation of activity for each region of the brain between two contiguous epochs of time averaged across all regions:
  1 $$S_L = \frac{1}{{\left( {n - 1} \right)}}\left( {r\left\{ {t_1,t_2} \right\},r\left\{ {t_2,t_3} \right\}, \ldots ,r\left\{ {t_{n - 1},t_n} \right\}} \right)$$
  whereby $r\{ {t_i,t_j}\}$ represents the correlation of activity across each region of the brain between two contiguous epochs of time (*i* and *i* + 1) – more stationary brain states/fewer variations of brain state configurations between time points have a higher value (and vice versa);
2. Global similarity (*S*_*G*_): the mean correlation of brain state configurations between any two points in time across the duration of the scan (as opposed to contiguous points to differentiate it from *S*_*L*_):
  2 $$S_G = \frac{1}{{n\left( {n - 1} \right)}}\left( {\mathop {\sum}\nolimits_{\scriptstyle i = 1\hfill \atop\\ \scriptstyle j = 1\hfill }^n {\left| {r\left\{ {t_i,t_j} \right\}} \right| - n} } \right)$$
  where $r\left\{ {t_i,t_j} \right\}$ represents the correlation between two time points *i* and *j*, which is summed over all possible time points *n*.

Accordingly, *S*_*L*_ quantifies the stationarity of brain state configurations for a given patient over time such that more stationary brain states (fewer variations of brain state configurations between time points) have a higher value of *S*_*L*_ and vice versa. Meanwhile, *S*_*G*_ can be used as a measure of the repertoire of brain states exhibited over the course of the scan so that subjects with minimally varying or frequently recurring states would have a higher *S*_*G*_ (closer to 1) than instances in which brain states change substantially (closer to 0).

To validate our measures, we calculated the similarity statistics in a separate cohort of 477 healthy controls obtained from the human connectome project and compared them to those from our present control sample (https://www.humanconnectome.org; Supplementary Table 3).

### Time resolved community and hub structure

Time-averaged and time-resolved community structure was calculated using the Brain Connectivity Toolbox^60^ (available from https://sites.google.com/site/bctnet/). Within-module connectivity was estimated by calculating the time-resolved module-degree *Z* score (W_*T*_) for each parcel^61^. Between-module connectivity was determined by the participation coefficient (*B*_*T*_) which represents the extent to which a region is connected across all modules relative to its connections within any single module. The participation coefficient of a region is close to 1 if its connections are uniformly distributed among all the modules and 0 if all its links are within one module. Details of these calculations are provided in the Supplementary Note 1.

### Characterisation of network topology

To track fluctuations in network topology over time, for each temporal window, we computed a 2D histogram of within- and between-module connectivity measures across all regions (referred to as a ‘cartographic profile’)^16,20^. The cartographic profile is a group-level joint histogram of the time-resolved *B*_T_ and *W*_T_ scores for each region. Colour intensity at any point reflects the cumulative sum of regions traversing the same topological state as defined by the two dimensions of between- and within-module connectivity (*B*_T_ and *W*_T_, respectively). The cartographic profile provides information about the global topological state of the network with the more leftward aspect of the cartographic profile representing more segregated states, and rightward displacement suggesting a more integrated profile. Code for this analysis is freely available at https://github.com/macshine/integration.

### Regional modular instability (flexibility)

The modular instability (flexibility) of each brain parcel was calculated by the percentage of temporal windows in which an individual region ‘switched’ between modules, normalised to the total number of modules in the data (as estimated in the previous step)^62^. As the modular assignment was essentially arbitrary within each unique temporal window, we used a version of the Hungarian algorithm to assign regions to modules with consistent values over time^11^.

### Sustained attention response task

Participants underwent attentional testing outside the scanner using the Sustained Attention Response Task (SART)^63,64^. The SART is a simple and ecologically valid task designed to measure failures in sustained attention and is based on the Go/No-Go paradigm with a high ratio of go trials to no-go trials^65,66^. We have shown that the SART can be applied in DLB populations and relate to cognitive fluctuations^64,67,68^. As the version of the SART used here was optimised to extract meaningful variance in DLB subjects^64^, we observed a ceiling effect of performance in control participants with the SART (accuracy 98.7 ± 1.2%) which precluded meaningful correlation with imaging measures in the control group.

To deconstruct SART performance into its underlying neurocognitive processes and account for speed-accuracy trade-offs, we fitted a drift diffusion model to each subject’s mean RT, RT variance and accuracy^69^. The model output included the psychologically relevant parameters representing the speed and accuracy of information processing (drift rate), the contribution of perceptual and motor processes not directly related to the decision process (non-decision time) and a flexible measure of response caution (boundary separation)^70^.

### Neurotransmitter/neuromodulator receptor gene expression

Gene expression profiles for cholinergic (nicotinic and muscarinic), dopaminergic and noradrenergic receptors were obtained using a spatial map of transcriptomic data derived from the Allen Human Brain Atlas^71^. In this study, we used a validated spatial map of predicted mRNA expression profiles of selected genes that has been registered to MNI space^72^. A full list of pre-selected genes is shown in Supplementary Table 2. Mean regional expression levels of these genes were obtained based on the parcellation used in our imaging analysis above. Spearman correlations were carried out between regional differences in participation (*B*_*T*_) or flexibility and the mean expression level of the chosen genes (FDR < 0.1 – threshold chosen a priori as an acceptable trade-off between Type I and Type II errors). Genes showing significant differences at the whole-brain level were then used in the subsequent analysis comparing regions of significant difference between controls and DLB.

### Statistical analysis

Statistical analysis was performed using MATLAB (Release 2019b, The MathWorks, Inc. Massachusetts, United States). Demographic variables were compared with independent samples *t* tests and chi-squared tests, where appropriate. Summary measures of temporal correlations of brain-state configurations were compared statistically between groups using independent samples *t* tests. Group comparisons of network properties was performed using permutation testing (iterations = 5000), controlling for age and sex^73^. To determine whether there were any abnormalities in functional network topology between groups, the mean cartographic profile was compared between groups (independent-samples *t* test for each bin of the cartographic profile; FDR *q* ≤ 0.05). Pearson correlations were performed between imaging and attention variables with permutation testing in the DLB cohort (*P* < 0.05). Pearson correlations between subjective and objective attentional measures were also performed against the mean cartographic profile for each bin of the cartographic profile (reported for values with FDR *q* ≤ 0.05). Comparison of gene expression profiles between regions of significantly different participation or flexibility was conducted using independent samples *t* test (*P* < 0.05) after confirming assumptions for normality, correcting for multiple comparisons.

### Reporting summary

Further information on research design is available in the Nature Research Reporting Summary linked to this article.

## Supplementary information

Supplementary Material

Reporting Summary

## Data Availability

Data supporting the findings of this study are available from the corresponding author, upon reasonable request.
