# Supplementary material for: Dynamic network impairments underlie cognitive fluctuations in Lewy body dementia
Source: NPJ Parkinsons Dis. 2022 Feb 17;8:16. doi: 10.1038/s41531-022-00279-x (PMC8854384; doi:10.1038/s41531-022-00279-x)
Supplement: Supplementary file 1 — Supplementary Material [file 41531_2022_279_MOESM1_ESM.pdf]

Supplementary Note 1

Calculation of time-resolved community and hub-structure

The Louvain algorithm iteratively maximizes the modularity statistic, Q, for different community assignments until the maximum possible score of Q has been obtained. Calculation of the Modularity statistic Q_T:

$$Q_T = \frac{1}{v^+}\sum_{ij}\left(w_{ij}^+ - e_{ij}^+\right)\delta_{M_iM_j} - \frac{1}{v^+ + v^-}\sum_{ij}\left(w_{ij}^- - e_{ij}^-\right)\delta_{M_iM_j} \qquad (1)$$

Where Q_T is the modularity statistic at a specific timepoint, v^+ and v^- are the sum of all positive and negative connection weights, w_{ij} is the weighted and signed connection between regions i and j, e_{ij} is the strength of a connection divided by the total weight of the network, and δ_{MiMj} is set to 1 when regions are in the same community and 0 otherwise. The plus and minus sign symbols denote all positive and negative connections, respectively.

The modularity statistic for a given adjacency matrix quantifies the extent to which the network may be subdivided into communities with stronger within-module than between-module connections. Using this technique, time-averaged and time-resolved community structure was calculated for each participant.

For each temporal window, regional community assignment was assessed 100 times and a consensus partition was identified using a fine-tuning algorithm from the Brain Connectivity Toolbox. This then afforded an estimate of both the time resolved modularity (Q_T) and cluster assignment within each temporal window for each participant in the study. A γ parameter of 1.1 provided the most robust estimates of topology across these iterations (quantified by the minimum standard deviation across 100 iterations of the Louvain algorithm).

Based on time-resolved community assignments, we estimated within-module connectivity by calculating the time-resolved module-degree Z-score (W_T) for each parcel (31):

$$W_{iT} = \frac{\kappa_{iT} - \bar{\kappa}_{s_{iT}}}{\sigma_{\kappa_{s_{iT}}}} \quad (2)$$

Module degree Z-score, W_{iT}, where κ_{iT} is the strength of the connections of region *I* to other regions in its module s_i at time *T*, κ_{s_iT} is the average of κ over all the regions in s_i at time *T*, and $\sigma_{\kappa s_iT}$ is the standard deviation of κ in s_i at time *T.*

Between-module connectivity was determined by the participation coefficient, B_T, which represents the extent to which a region is connected across all modules relative to its connections within any single module:

$$B_{iT} = 1 - \sum_{s=1}^{n_M} \left(\frac{\kappa_{isT}}{\kappa_{iT}}\right)^2 \quad (3)$$

Participation coefficient B_{iT}, where κ_{isT} is the strength of the positive connections of region *I* to regions in module *s* at time *T*, and κ_{iT} is the sum of strengths of all positive connections of region *I* at time *T.* The participation coefficient of a region is close to 1 if its connections are uniformly distributed among all the modules and 0 if all its links are within one module.

Supplementary Table 1

Supplementary Table 1. Correlation coefficients between time-resolved imaging statistics and behavioural measures in DLB participants (n=22)

	**Time-resolved measures**	
**Behavioural measures**	**Local similarity (S_L)**	**Global similarity (S_G)**
**SART**		
Accuracy (%)	-0.221	0.111
RT (mean)	0.144	0.066
RT (SD)	**0.426***	-0.017
Drift rate	**-0.414***	0.025
Decision threshold	0.224	0.002
Non-decision time	-0.419	-0.152
MMSE score	-0.321	0.148

*p<0.05, randomized permutation testing (5000 iterations); RT=response time; SD=standard deviation.

Supplementary Table 2

Supplementary Table 2. List of selected genes analysed from the Allen Human Brain Atlas

Ligand	Gene Symbol	Receptor (Subunit) Name
Norepinephrine	*ADRA1A*	Alpha-1A adrenergic receptor
	ADRA1B	Alpha-1B adrenergic receptor
	ADRA1D	Alpha-1D adrenergic receptor
	ADRA2A	Alpha-2A adrenergic receptor
	ADRA2B	Alpha-2B adrenergic receptor
	ADRA2C	Alpha-2C adrenergic receptor
Acetylcholine	*CHRM1*	Muscarinic Acetylcholine Receptor M1
	CHRM2	Muscarinic Acetylcholine Receptor M2
	CHRM3	Muscarinic Acetylcholine Receptor M3
	CHRM4	Muscarinic Acetylcholine Receptor M4
	CHRM5	Muscarinic Acetylcholine Receptor M5
	CHRNA1	Nicotinic Cholinergic Receptor (Alpha 1)
	CHRNA2	Nicotinic Cholinergic Receptor (Alpha 2)
	CHRNA3	Nicotinic Cholinergic Receptor (Alpha 3)
	CHRNA4	Nicotinic Cholinergic Receptor (Alpha 4)
	CHRNA5	Nicotinic Cholinergic Receptor (Alpha 5)
	CHRNA6	Nicotinic Cholinergic Receptor (Alpha 6)
	CHRNA7	Nicotinic Cholinergic Receptor (Alpha 7)
	CHRNA9	Nicotinic Cholinergic Receptor (Alpha 9)
	CHRNA10	Nicotinic Cholinergic Receptor (Alpha 10)
	CHRNB1	Nicotinic Cholinergic Receptor (Beta 1)
	CHRNB2	Nicotinic Cholinergic Receptor (Beta 2)
	CHRNB3	Nicotinic Cholinergic Receptor (Beta 3)
	CHRND	Nicotinic Cholinergic Receptor (Delta)
	CHRNE	Nicotinic Cholinergic Receptor (Epsilon)
Dopamine	*DRD1*	Dopamine Receptor D1
	DRD2	Dopamine Receptor D2
	DRD3	Dopamine Receptor D3
	DRD4	Dopamine Receptor D4
	DRD5	Dopamine Receptor D5

Supplementary Table 3

Supplementary Table 3. Clinical characteristics of the original study controls and validation dataset of controls derived from the Human Connectome Project (HCP)

	Discovery (original)	Validation dataset (HCP)
N	49	477
Sex (F)	14:35	18:4
Age	66.4 (8.5)	31 (3.9)
MMSE	28.9 (1.2)	29.1 (1.0)

Values presented as mean (standard deviation) unless otherwise specified as number of patients (percentages).

Supplementary Figure 1

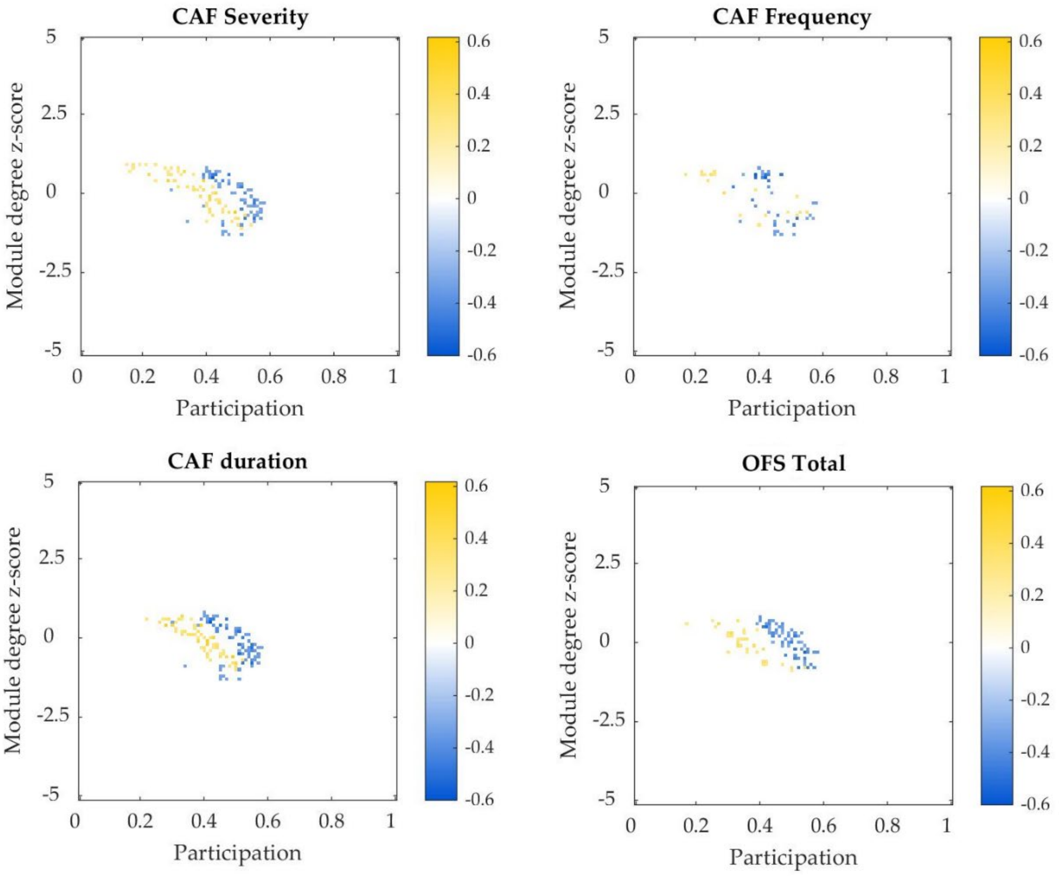

Supplementary Figure 1. Correlation between the cartographic topological profile and subjective reporting of fluctuations. The cartographic profile is a histogram mapping the functional activity of each parcellated region of the brain according to the topological dimensions of between-module connectivity (participation) and within-module connectivity (module degree z-score). Correlations displayed for regions with FDR<0.2. More integrated regions can be seen to negatively map to higher CAF Severity and Duration scores and OFS scores while the converse is true for regions of segregation. No clear pattern is seen for reported frequency of fluctuations. Colour bar denotes Spearman's correlation coefficient. CAF=Clinician Assessment of Fluctuations Scale; OFS = One Day Fluctuation Scale.
