# Supplementary material for: Dynamic network impairments underlie cognitive fluctuations in Lewy body dementia
Source: NPJ Parkinsons Dis. 2022 Feb 17;8:16. doi: 10.1038/s41531-022-00279-x (PMC8854384; doi:10.1038/s41531-022-00279-x)
Supplement: Supplementary file 2 — Reporting Summary [file 41531_2022_279_MOESM2_ESM.pdf]

nature portfolio

Corresponding author(s): Elie Matar

Last updated by author(s): Aug 11, 2021

Reporting Summary

Nature Portfolio wishes to improve the reproducibility of the work that we publish. This form provides structure for consistency and transparency in reporting. For further information on Nature Portfolio policies, see our Editorial Policies and the Editorial Policy Checklist.

Statistics

For all statistical analyses, confirm that the following items are present in the figure legend, table legend, main text, or Methods section.

n/a	Confirmed	
☐	☒	The exact sample size (*n*) for each experimental group/condition, given as a discrete number and unit of measurement
☐	☒	A statement on whether measurements were taken from distinct samples or whether the same sample was measured repeatedly
☐	☒	The statistical test(s) used AND whether they are one- or two-sided<br>*Only common tests should be described solely by name; describe more complex techniques in the Methods section.*
☐	☒	A description of all covariates tested
☐	☒	A description of any assumptions or corrections, such as tests of normality and adjustment for multiple comparisons
☐	☒	A full description of the statistical parameters including central tendency (e.g. means) or other basic estimates (e.g. regression coefficient) AND variation (e.g. standard deviation) or associated estimates of uncertainty (e.g. confidence intervals)
☐	☒	For null hypothesis testing, the test statistic (e.g. *F*, *t*, *r*) with confidence intervals, effect sizes, degrees of freedom and *P* value noted<br>*Give P values as exact values whenever suitable.*
☐	☒	For Bayesian analysis, information on the choice of priors and Markov chain Monte Carlo settings
☐	☒	For hierarchical and complex designs, identification of the appropriate level for tests and full reporting of outcomes
☐	☒	Estimates of effect sizes (e.g. Cohen's *d*, Pearson's *r*), indicating how they were calculated

Our web collection on statistics for biologists contains articles on many of the points above.

Software and code

Policy information about availability of computer code

Data collection	Microsoft Excel 2016
Data analysis	MATLAB (Release 2019b, The MathWorks, Inc. Massachusetts, United States

For manuscripts utilizing custom algorithms or software that are central to the research but not yet described in published literature, software must be made available to editors and reviewers. We strongly encourage code deposition in a community repository (e.g. GitHub). See the Nature Portfolio guidelines for submitting code & software for further information.

Data

Policy information about availability of data

All manuscripts must include a data availability statement. This statement should provide the following information, where applicable:

- Accession codes, unique identifiers, or web links for publicly available datasets
- A description of any restrictions on data availability
- For clinical datasets or third party data, please ensure that the statement adheres to our policy

Data supporting the findings of this study are available from the corresponding author, upon reasonable request.

Field-specific reporting

Please select the one below that is the best fit for your research. If you are not sure, read the appropriate sections before making your selection.

☒ Life sciences ☐ Behavioural & social sciences ☐ Ecological, evolutionary & environmental sciences

For a reference copy of the document with all sections, see nature.com/documents/nr-reporting-summary-flat.pdf

Life sciences study design

All studies must disclose on these points even when the disclosure is negative.

Sample size	Sample sizes reflect similar sample sizes in previous functional neuroimaging studies that have demonstrated significant differences between patient populations and controls. Also, as we were looking at biomarkers, significant results relying on large effect sizes were desirable. Given the modest sample size for the genetic associations, these were presented as an exploratory analysis.
Data exclusions	Control participants were excluded if they had a history of neurological or psychiatric disorders or had a prescription for psychoactive medications. Patients (n=3) who reported falling asleep were excluded from the analysis.
Replication	To support our findings, we applied our statistical measures to a a validation sample of 477 controls derived from the human connectome project. Findings demonstrated no significant difference between our sample and the validation dataset (SL= 0.48 ± 0.1, P=0.37; SG= 0.21 ± 0.07, P=0.46; two-sided independent samples t-test).
Randomization	Case control / cross-sectional study design. Consecutive cases with DLB were used. Controls were recruited from a clinical setting and age-matched.
Blinding	Blinding was not relevant to the study as the study investigators were required to be involved in the data collection. The hypothesis and analysis method was unlikely to be affected by lack of blinding.

Reporting for specific materials, systems and methods

We require information from authors about some types of materials, experimental systems and methods used in many studies. Here, indicate whether each material, system or method listed is relevant to your study. If you are not sure if a list item applies to your research, read the appropriate section before selecting a response.

Materials & experimental systems

n/a	Involved in the study	
☒	☐	Antibodies
☒	☐	Eukaryotic cell lines
☒	☐	Palaeontology and archaeology
☒	☐	Animals and other organisms
☐	☒	Human research participants
☒	☐	Clinical data
☒	☐	Dual use research of concern

Methods

n/a	Involved in the study	
☒	☐	ChIP-seq
☒	☐	Flow cytometry
☐	☒	MRI-based neuroimaging

Human research participants

Policy information about studies involving human research participants

Population characteristics	N -Controls 49 DLB 22<br>Sex (M:F) * Controls - 14:35 DLB - 18:4<br>Age - Controls - 66.4 (8.5), DLB - 74.5 (6.1)<br>Education - Controls - 13.5 (2.8), DLB - 12.0 (3.3)<br>MMSE * Controls - 28.9 (1.2), DLB - 22.7 (5.7)
Recruitment	participants were prospectively and consecutively recruited from a community dwelling population referred to a dementia and movement disorders clinic at the Brain and Mind Centre, University of Sydney. Control participants were recruited from the community via use of flyers, online advertising, word-of-mouth, email to previous study participants who have opted to be contacted for future studies and recruitment drives at local facilities e.g. golf course. The recruitment method could be subject to selection bias - recruiting more severe patients, or a specific subgroup of controls who participate in studies (i.e. may be more cognitively healthy).
Ethics oversight	University of Sydney Human Ethics Research Committee

Note that full information on the approval of the study protocol must also be provided in the manuscript.

Magnetic resonance imaging

Experimental design

Design type	Resting state
Design specifications	Resting T2*-weighted echo planar functional images were acquired in interleaved order (repetition time=3 s, echo time=36 ms, flip angle=90°, 40 axial slices, field of view = 240 mm, raw voxel size = 3.75 × 3.75 × 3 mm thick, duration=7 minutes).
Behavioral performance measures	Patients were instructed to lie awake with their eyes closed and to let their minds wander freely without falling asleep. Patients were prompted prior to the fMRI sequence and interviewed after the sequence and scan to ensure these instructions were followed. Patients (n=3) who reported falling asleep were excluded from the analysis.

Acquisition

Imaging type(s)	Functional
Field strength	3T
Sequence & imaging parameters	Whole brain T1 - 200 slices, 1 × 1 mm2 in-plane resolution, flip angle 12°, slice thickness=1 mm, echo time/repetition time = 2.7/7.1 ms.<br>Resting T2*-weighted echo planar functional images were acquired in interleaved order (repetition time=3 s, echo time=36 ms, flip angle=90°, 40 axial slices, field of view = 240 mm, raw voxel size = 3.75 × 3.75 × 3 mm thick, duration=7 minutes
Area of acquisition	Whole brain

Diffusion MRI ☐ Used ☒ Not used

Preprocessing

Preprocessing software	SPM12 (Statistical Parametric Mapping software; http://www.fil.ion.ucl.ac.uk/spm/software/).
Normalization	Affine transform followed by a nonlinear registration of the EPI image to an EPI template in standard space (MNI)
Normalization template	Images were normalized to the Echo Planar Image template in MNI space and resampled to 3 mm isotropic voxels.
Noise and artifact removal	None of the subjects included in this study demonstrated scan-to-scan head movements >3 mm (<1 voxel breadth). Temporal artefacts were identified in each dataset by calculating framewise displacement (FD) from the derivatives of the six rigid-body realignment parameters estimated during standard volume realignment, as well as the root mean square change in BOLD signal from volume to volume (DVARS). Frames associated with FD > 0.25 mm or DVARS > 2.5% were identified. However, as no participants were identified with >10% of the resting time points exceeding these values, no sessions were excluded from further analysis. Nuisance covariates associated with the 12 linear head movement parameters (and their temporal derivatives), FD, DVARS, and anatomical masks from the CSF and deep cerebral white matter were regressed from the data using the aCompCor strategy.
Volume censoring	No volume censoring was performed

Statistical modeling & inference

Model type and settings	Dynamic sliding window functional connectivity analyses were performed and graph theoretical measures of connectivity (as detailed in the Methods and Supplementary Materials) were derived an applied to the functional connectivity matrices applied to each window of time.
Effect(s) tested	Participation coefficient, modularity, module degree z-score, cartographic analysis

Specify type of analysis: ☒ Whole brain ☐ ROI-based ☐ Both

Statistic type for inference<br>(See Eklund et al. 2016)	Permutation testing based on graph theoretical measures derived from functional connectivity data.
Correction	FDR

Models & analysis

n/a	Involved in the study
☐	☒ Functional and/or effective connectivity
☐	☒ Graph analysis
☒	☐ Multivariate modeling or predictive analysis

Functional and/or effective connectivity	Pearson correlation
Graph analysis	Weighted
